# Supplementary material for: Integrase-Defective Lentiviral Vectors for Delivery of Monoclonal Antibodies against Influenza
Source: Viruses. 2020 Dec 17;12(12):1460. doi: 10.3390/v12121460 (PMC7767071; doi:10.3390/v12121460)
Supplement: Supplementary file 1 [file viruses-12-01460-s001.pdf]

viruses

MDPI

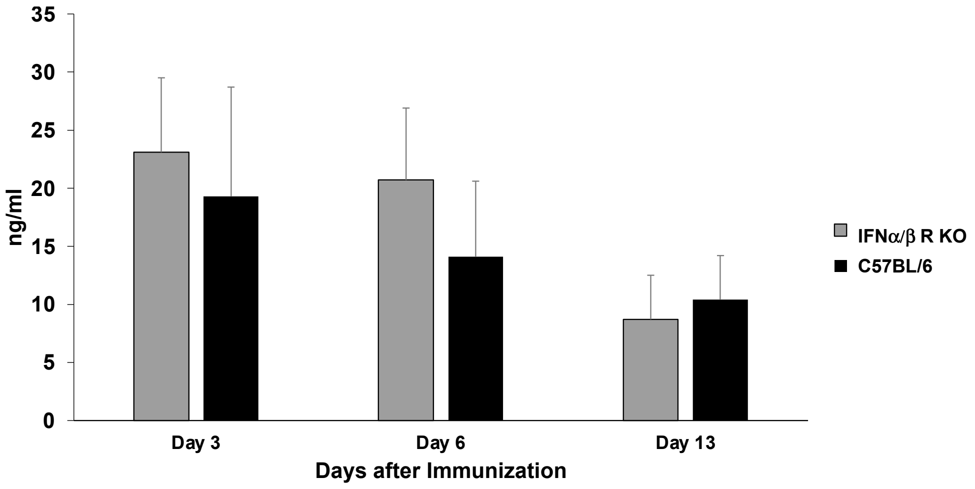

Figure 1. Production of mAbs in C57BL/6 and IFNα/β receptor knock-out mice after IDLV-VN04-2 administration in vivo. Groups of 5 C57BL/6 and IFNα/β receptor knock-out (IFNα/β R KO) mice were given IDLV-VN04-2 (about 400 ng RT/mouse) by the i.n. route of administration. The production of VN04-2 mAb in the serum was measured by ELISA for human IgG at different points post IDLV-VN04-2 administration, as indicated in the figure.

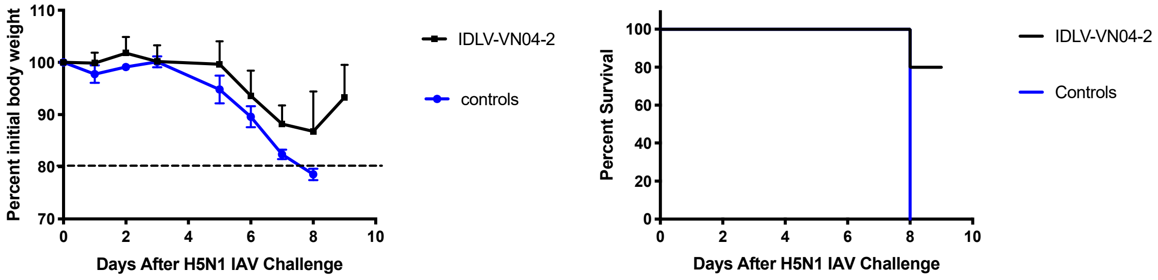

Figure S2. Protection from H5N1 IAV challenge at 21 days after IDLV-VN04-2 administration. Groups of 5 BALB/c mice received either IDLV-VN04-2 by the i.n. route (about 160 ng RT/mouse) or were left unimmunized as a control. Mice were then lethally challenged with VNH5N1-PR8 IAV at 21 days after IDLV administration. Weight loss (**a**) and survival (**b**) are shown. Mice with a weight loss of ≥20% of their initial body weight were euthanized and counted as dead (Comparison of survival curves, $p = 0.02$, Log-rank Mantel-Cox test).

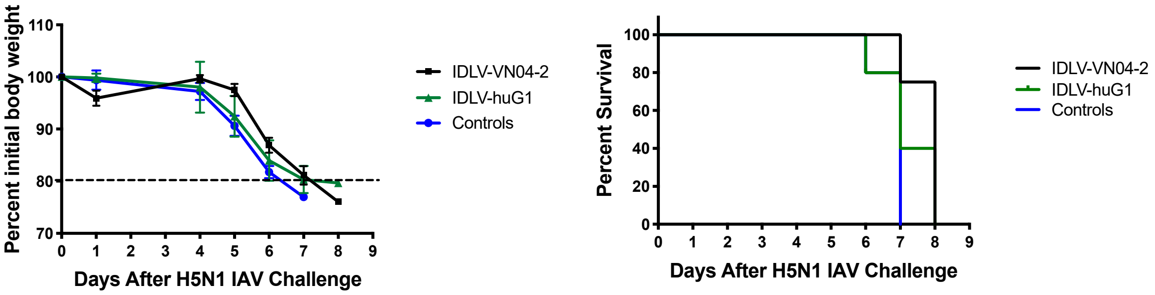

(a) (b)

Figure S3. IDLV-VN04-2 does not protect from H5N1 IAV challenge at 30 days after administration. Groups of 5 BALB/c mice received either IDLV-VN04-2 or IDLV-huG1 as a control by the i.n. route (about 250ng RT/mouse), or were left unimmunized. Mice were then lethally challenged with VNH5N1-PR8 IAV at 30 days after IDLV administration. Weight loss (**a**) and survival (**b**) are shown. Mice with a weight loss of ≥20% of their initial body weight were euthanized and counted as dead. Data representative of two separate experiments are shown (comparison of survival curves p = not significant, Log Rank Mantel-Cox test).

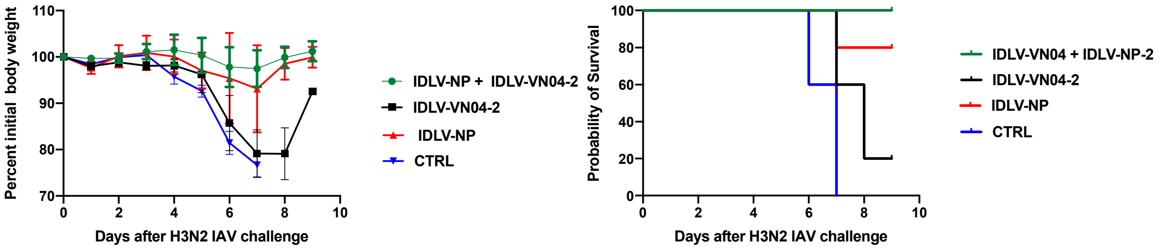

(a) (b)

Figure S4. Combined administration of ILDV-VN04-2 and IDLV-NP protects from H3 IAV challenge Groups of 5 BALB/c mice received IDLV-VN04-2, IDLV-NP or a combination of IDLV-VN04-2 and IDLV-NP by the i.n. route (approximately 300 ng of each vector). Mice left unimmunized were used as a control. Since IDLV-NP is unable to protect after a single administration, mice who had received IDLV-NP alone or in combination received a second dose of this vector 14 days after the first administration. All mice were then lethally challenged with IAV Phil/H3N2 30 days after the initial IDLV administration. Weight loss (**a**) and survival (**b**) are shown. Mice receiving the combination of IDLV-VN04-2 and IDLV-NP, but not IDLV-VN04-2 alone, were protected from challenge, suggesting that IDLV-VN04-2 does not interfere with IDLV-NP-induced protection. (comparison of the survival curves: p = 0.014, Log-rank Mantel-Cox test).
